# Evaluating the Efficacy of Body-First Technique Compared to the Conventional Technique in Achieving a Critical View of Safety in Laparoscopic Cholecystectomy: A Randomized Controlled Trial

**DOI:** 10.7759/cureus.111629

**Published:** 2026-06-27

**Authors:** Ravi Gupta, Jagani Harsha Sai, Gaurav Gupta, U Venkatesh, Dharmendra Kumar Pipal, Mukul Singh, Raja Murtaza, Tanushree Trivedi, Rajesh Kannan, Darshan Jalwal

**Affiliations:** 1 General Surgery, All India Institute of Medical Sciences, Gorakhpur, Gorakhpur, IND; 2 Community and Family Medicine, All India Institute of Medical Sciences, Gorakhpur, Gorakhpur, IND

**Keywords:** body-first technique, critical view of safety, gallstones, laparoscopic cholecystectomy, minimally invasive surgery

## Abstract

Background: Laparoscopic cholecystectomy (LAP-C) is the definitive treatment for symptomatic cholelithiasis, yet bile duct injury (BDI), primarily caused by anatomical misidentification, remains a formidable risk. The Critical View of Safety (CVS) achievement will prevent BDI, but its consistent attainment is technically demanding in inflamed or distorted anatomy. The body-first dissection technique has been proposed as an alternative approach to improve CVS achievement, though high-quality prospective evidence remains limited. This randomized controlled trial (RCT) was designed to systematically compare the two methods.

Methods: In this single-center, single-blinded RCT, 88 patients undergoing elective LAP-C were enrolled, after exclusion 80 patients were randomized into two groups: Group A (body-first technique) and Group B (conventional technique). The primary endpoints were the rate and quality of CVS achievement, and the duration of surgery. Secondary outcomes included intraoperative complications, recovery of gastrointestinal function, postoperative pain and nausea, inflammatory marker C-reactive protein (CRP) levels, and patient satisfaction assessed by the RAND-36 questionnaire at discharge, one month, and three months, and a p-value < 0.05 was considered statistically significant.

Results: The body-first approach demonstrated a higher rate of satisfactory CVS than the conventional approach (92.5% vs. 75.0%); however, the difference did not reach statistical significance on two-sided Fisher's exact testing (p=0.066). On multivariable logistic regression, the conventional technique was independently associated with significantly lower odds of CVS achievement (adjusted odds ratio (OR) 0.10, 95% CI 0.02-0.66, p=0.017). Operative duration was comparable between groups (76.3 vs. 84.8 minutes, p=0.438). After multivariable adjustment, surgical technique was not independently associated with operative duration (β = 6.54 minutes, SE=6.48; p=0.316), whereas increasing operative difficulty (Modified Nassar grade) was the only independent predictor of longer operative duration (β=13.32 minutes per one-grade increase, 95% CI 7.11-19.53; p < 0.001). The body-first group had significantly lower postoperative pain (OR: 5.74, 95% CI: 2.15-15.3, p=0.001), faster gastrointestinal recovery (OR: 3.12, 95% CI: 1.25-7.75, p=0.014).

Conclusion: The body-first technique confers a higher rate of achieving CVS, promotes faster gastrointestinal recovery, and reduces postoperative pain compared to the conventional technique. The benefits in safety and recovery make it a favorable alternative to the conventional technique in LAP-C.

## Introduction

Laparoscopic cholecystectomy (LAP-C) is the standard surgical treatment for symptomatic gallstone disease. It has largely replaced open cholecystectomy because of reduced postoperative pain, shorter hospital stays, earlier return to normal activity, and lower wound morbidity [1]. It was first performed in 1985 and rapidly disseminated globally, with India adopting it early by 1991 [2,3]. Despite these advantages, bile duct injury (BDI) remains one of the most feared complications of LAP-C, with an incidence reported between 0.3% and 0.7%, and is associated with significant long-term morbidity, medico-legal consequences, and the need for complex hepatobiliary reconstruction [4,5]. The predominant mechanism underlying BDI is misidentification of biliary anatomy rather than technical incompetence [2].

To address this problem, the Critical View of Safety (CVS) was introduced by Steven M. Strasberg as a structured method for ensuring correct anatomical identification before dividing cystic structures [6]. CVS requires complete clearance of the hepatocystic triangle of fat and fibrous tissue, separation of the lower third of the gallbladder from the cystic plate, and confirmation that only two tubular structures enter the gallbladder. International hepatobiliary societies now strongly advocate routine documentation of CVS, including photographic recording, as a quality and safety measure [7,8].

However, achieving CVS may be technically challenging in cases of inflammation, dense adhesions, contracted gallbladder, or aberrant anatomy [9]. The conventional technique begins with the dissection of Calot’s triangle, followed by early identification of the cystic duct and artery. In difficult anatomy, this may increase the risk of visual misperception and vasculobiliary injury [10]. The body-first approach initiates dissection at the gallbladder body, progressing retrogradely toward the hepatocystic triangle, potentially improving exposure and reducing early manipulation of inflamed structures [11-13]. Recent studies on capsule-based body-first methods, such as Laennec's membrane approach, have shown improved Calot's exposure and reduced vasculobiliary risks in inflamed cases by creating a clear subserosal plane prior to Calot's triangle dissection [14]. In the present scenario, surgeons usually perform LAP-C via a conventional technique; in the case of a frozen Calot's, a body-first approach is considered one of the bailout procedures.

Existing research on the body-first approach remains exceedingly limited, consisting primarily of retrospective observations and procedural reports, with virtually no high-quality prospective randomized controlled trials that provide direct comparisons to conventional methods regarding CVS achievement and comprehensive recovery parameters [11]. Addressing this critical deficit, especially the absence of randomized controlled trials from elective cases in the Indian surgical setting, this single-blinded randomized controlled trial was undertaken.

Our study's primary innovation is the blinded photographic assessment of CVS using standardized doublet documentation, enabling objective comparison between body-first and conventional approaches [15]. Unlike prior retrospective reports, we evaluated multiple endpoints, including CVS achievement, operative time, postoperative pain, gastrointestinal recovery, C-reactive protein (CRP) levels, and RAND-36 satisfaction scores up to three months. The randomized controlled trial (RCT) design with allocation concealment minimizes bias, while inclusion of diverse patients provides practical insights for routine LAP-C practice.

Drawing from pathophysiological rationale and preliminary studies, we hypothesized that the body-first technique would yield higher CVS achievement rates (primary endpoint) without prolonging operative time. We anticipated reduced postoperative pain, faster gastrointestinal recovery, and improved patient satisfaction in the body-first group, while expecting comparable complication rates, CRP levels, and hospital stays between groups. This would affirm body-first as a safe primary or bailout strategy, optimizing outcomes without added risk.

## Materials and methods

Study design and participants

After approval from the Institutional Human Ethics Committee at the All India Institute of Medical Sciences, Gorakhpur, India (IHEC/AIIMS-GKP/BMR/306/2024), this RCT was conducted between October 2024 and July 2025. This trial protocol was prospectively registered with the Clinical Trials Registry-India (CTRI) prior to participant enrolment (CTRI/2024/10/075571). The study protocol is available from the corresponding author upon reasonable request. No important changes were made to the study protocol, outcomes, or statistical analyses after trial commencement. Patients and the public were not involved in the design, conduct, reporting, or dissemination plans of this study.

Patients aged 18 years or older scheduled for elective LAP-C for symptomatic cholelithiasis, as verified by clinical assessment and imaging, were included after written informed consent was obtained. Exclusion encompassed individuals requiring emergency/urgent intervention for acute cholecystitis; those with prior major upper abdominal operations causing anatomical distortion (such as gastric bypass or hepatic resection); cases with confirmed or suspected biliary anomalies or ductal disorders (including Mirizzi syndrome, choledocholithiasis, or aberrant ductal patterns); contraindications to laparoscopy (e.g., severe cardiopulmonary compromise or uncorrected coagulopathy); pregnant women; patients on immunosuppressive agents within the prior month; and subjects with cognitive limitations preventing valid consent. The present study adhered to the Consolidated Standards of Reporting Trials (CONSORT) 2025 guidelines for the conduct and reporting of randomized controlled trials [16].

Sample size calculation

Based on previous research by Matsumura M et al., we calculated the sample size for a median difference in operative duration using G*Power® software 3.1.9.7 (Heinrich-Heine-Universität, Düsseldorf, Germany). Using t-test analysis for independent groups with effect size (Cohen's d) = 0.64, alpha error (α) = 0.05, power (1-β) = 0.80, and allocation ratio 1:1, the required sample size was calculated as 40 participants per group (total 80). Actual power achieved was approximately 80.7% [11]. No interim analyses or stopping guidelines were planned, given the relatively short study duration and the predefined sample size.

Randomization and blinding

Participants meeting the inclusion criteria provided written informed consent before enrolment. Randomization used computer-generated block randomization (blocks of 10, 5:5 allocation) to ensure equal probability of assignment to either group. An independent administrator prepared sealed, opaque, sequentially numbered envelopes (SNOSE) containing allocation information. Envelopes were opened after baseline assessments were complete and prior to surgical intervention. Patients were allocated to either Group A (body-first technique) or Group B (conventional technique) in a 1:1 ratio. Due to the nature of surgical techniques, surgeons could not be blinded to intervention assignment, but outcome assessors evaluating photographic documentation of CVS were blinded to group allocation.

Data collection and baseline assessment

Preoperative data included demographics (age, gender, body mass index), American Society of Anaesthesiologists (ASA) grade, clinical presentation, comorbidities, and relevant biochemical and radiological parameters. Intraoperative data included operative difficulty grading, technique employed, operative duration, CVS achievement and grading, anatomical variations, and complications. Postoperative data included immediate and delayed complications, pain scores, recovery metrics, and patient-reported outcomes assessed at standardized intervals (discharge, one month, three months).

Surgical procedure

All operations were performed by experienced surgeons trained in both techniques under general anesthesia, using a standard laparoscopic instrument setup (4-port technique: 10-mm umbilical port for laparoscope, 5-mm anterior axillary port for retraction, 10-mm epigastric port for instrument passage, 5-mm subcostal port for dissection and hemostasis).

Group A: Body-First Technique

**Figure 1:**
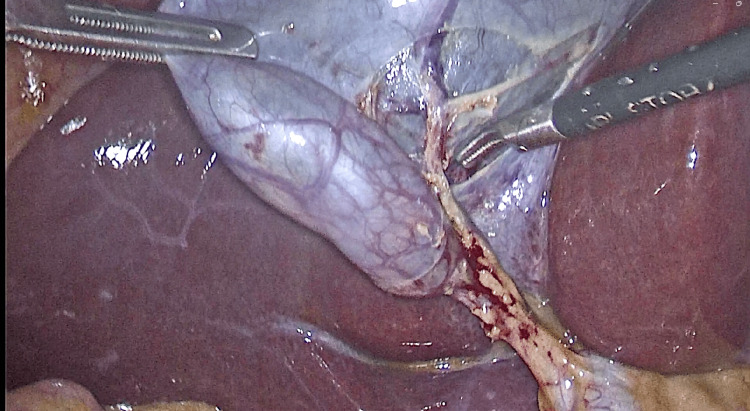
Intraoperative picture of body-first approach in laparoscopic cholecystectomy Dissection commenced at the gallbladder body, allowing development of the plane between the gallbladder and the cystic plate. This maneuver enabled effective retraction of the gallbladder away from critical vasculobiliary structures, contributing to a safer cholecystectomy.

Following diagnostic laparoscopy and operative difficulty grading (Modified Nassar scale), anterior and posterior peritoneal fold dissection was performed [5]. Dissection commenced at the gallbladder body in the subserosa plane between the cystic plate and gallbladder wall, proceeding retrogradely toward Calot's triangle without initially engaging cystic structures (Figure 1). This retrograde dissection created a window between the gallbladder and the hepatic hilum, allowing controlled mobilization and exposure of the hepatocystic triangle prior to cystic vessel identification. Once adequate exposure was achieved with clear visualization of anatomical planes, the cystic artery and duct were identified and divided after confirming CVS criteria.

Group B: Conventional Technique

**Figure 2:**
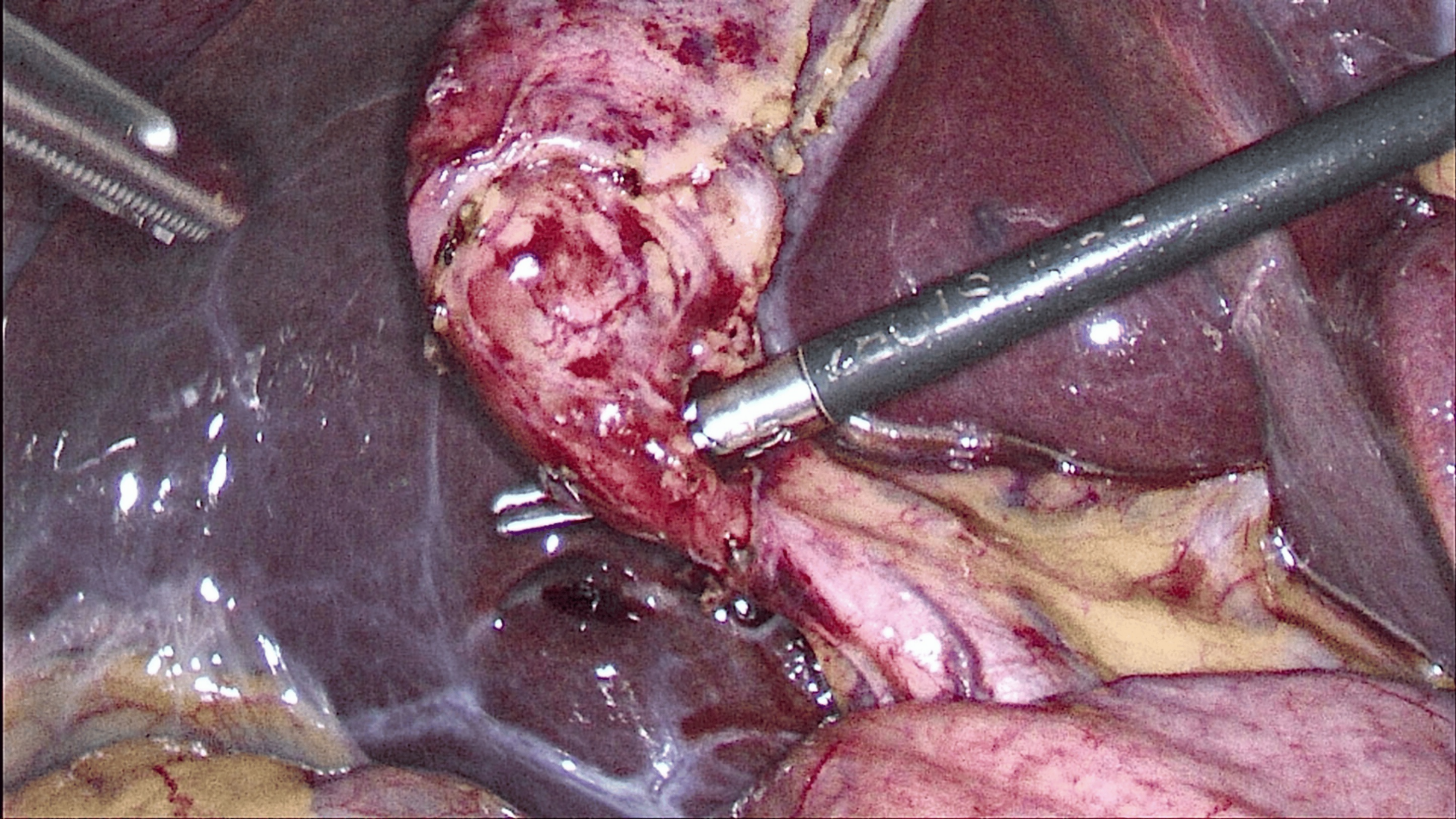
Intraoperative picture of conventional approach (cystic duct first) in laparoscopic cholecystectomy In the conventional approach, after proper retraction of Hartmann’s pouch, dissection was commenced in Calot’s triangle, and the cystic artery and cystic duct were dissected initially.

Following diagnostic laparoscopy and operative difficulty grading, anterior and posterior peritoneal fold dissection was performed [5]. Calot's triangle was dissected systematically with early identification and division of the cystic artery, followed by cystic duct identification and division (Figure 2). CVS was achieved once anatomical clarity was established.

Pre-dissection and post-dissection still photography of anterior and posterior views was performed after Hartmann's retraction using a 30-degree laparoscope with white balance optimization. Photographs captured the hepatocystic triangle demonstrating: complete clearance of fat and fibrous tissue, visualization of two tubular structures (cystic artery and duct), and clear visualization of the cystic plate with hepatocystic triangle separation. Photographic doublets were assessed by experienced surgeons blinded to group allocation using standardized scoring criteria (satisfactory = score ≥5; unsatisfactory = score <5 or failure to meet CVS criteria).

**Figure 3:**
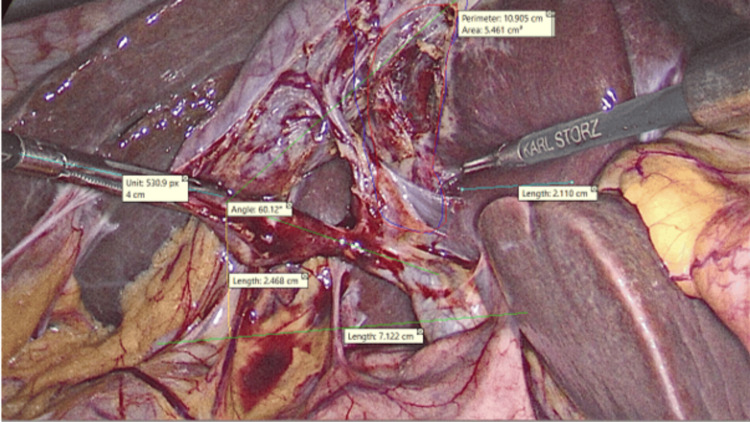
Intraoperative view depicting the end point of dissection in laparoscopic cholecystectomy The endpoint of dissection is characterized by attainment of the Critical View of Safety. Intraoperative objective assessment of the achievement of the critical view of safety was performed using Digimizer® software (MedCalc Software Ltd, Ostend, Belgium), measuring the critical angle and critical area.

The cystic duct fold (critical angle) was calculated from doublet photographs using Digimizer® software, version 6.5.0 (MedCalc Software Ltd, Ostend, Belgium), measuring the peritoneal fold stretching between the retracted Hartman's pouch and cystic duct. Pre-dissection and post-dissection angles were measured, and the ratio was correlated with CVS quality (Figure 3).

All participants received identical perioperative and postoperative care according to institutional protocols, including standardized anesthetic management, antibiotic prophylaxis, postoperative analgesia, early mobilization, and dietary advancement. No additional interventions that could influence the primary or secondary outcomes were administered differentially between groups.

Outcome measures

The primary endpoint was the rate and quality of CVS achievement (satisfactory/unsatisfactory per Strasberg criteria), with operative duration (skin-to-skin time in minutes) as the co-primary outcome [6,15]. Secondary endpoints encompassed intraoperative complications (bile duct injury, haemorrhage, gallbladder perforation, bowel trauma), open conversion rates and indications, postoperative pain assessed using a 10-cm Visual Analogue Scale (VAS), adverse events (wound infection, bile leak, fever), nausea/vomiting, time to first flatus (hours), 24-hour CRP levels, and health-related quality of life assessed using the RAND-36 questionnaire. RAND-36 assessments were performed at discharge, one month, and three months after surgery to evaluate early postoperative recovery, intermediate functional recovery, and short-term restoration of health-related quality of life, respectively [17].

Statistical analysis

The study’s data were analyzed using IBM SPSS Statistics for Windows, Version 26.0 (IBM, Armonk, NY, USA). Descriptive statistics, including means, standard deviations, medians, and percentages, summarized the study population. Categorical variables were compared using the Pearson chi-square test or Fisher's exact test, as appropriate. Fisher's exact test was applied when expected cell counts were less than five. The Mann-Whitney U test was used to compare continuous variables, such as operative duration, due to expected non-normal distributions. Postoperative pain scores and inflammatory markers were compared using t-tests or Mann-Whitney U tests, depending on data distribution. Multivariable logistic regression was performed to evaluate the independent association between surgical technique and satisfactory CVS achievement. Given the limited number of outcome events, the model was restricted to surgical technique and operative difficulty (Modified Nassar grade) to minimize overfitting. Adjusted odds ratios (ORs) with 95% confidence intervals (CIs) were reported. Before multivariable modeling, multicollinearity was assessed using variance inflation factors (VIF) and tolerance statistics, with no evidence of significant multicollinearity. There were no missing outcome data, and all randomized participants completed follow-up assessments. Two-sided p-values <0.05 were considered statistically significant.

Ethical considerations

The Institutional Human Ethics Committee at the All India Institute of Medical Sciences, Gorakhpur, approved the study protocol. All participants provided written informed consent after a detailed explanation of the study aims, procedures, risks, and benefits. Data were maintained confidentially with restricted access. Any adverse events were immediately reported to the IHEC with appropriate management and protocol adjustments as needed.

## Results

Baseline characteristics and demographics

**Figure 4:**
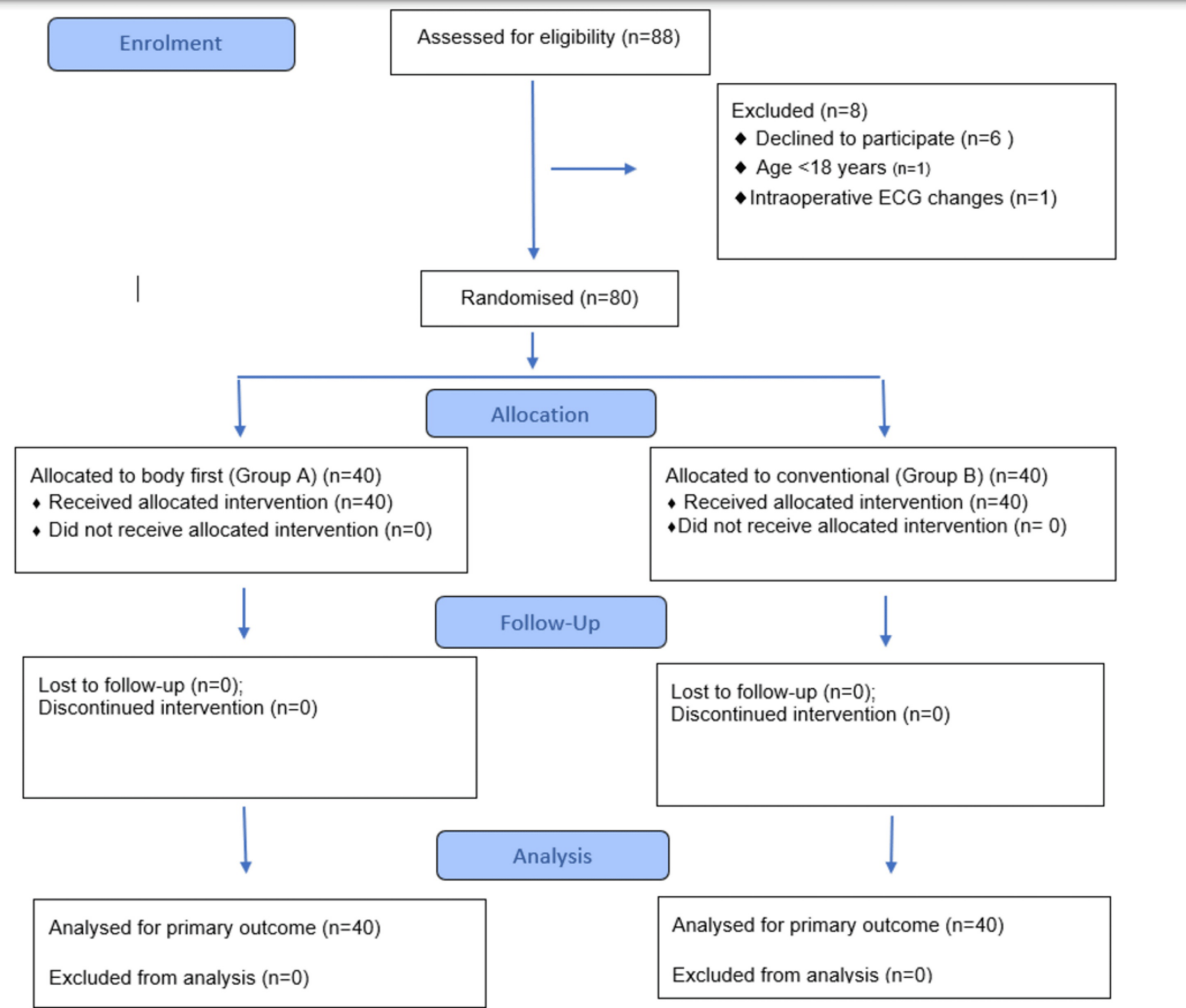
CONSORT flow diagram Flow diagram depicting participant screening, exclusion, randomization, allocation, follow-up, and final analysis in the randomized controlled trial comparing body-first versus conventional laparoscopic cholecystectomy techniques. CONSORT = Consolidated Standards of Reporting Trials.

A total of 80 patients undergoing LAP-C were randomized to two groups: 40 patients in the Body-First Approach group and 40 patients in the Conventional Approach group (Figure 4). The study was completed as planned and was not terminated or stopped early.

The study population comprised predominantly females (76.3%, n=61) with males accounting for 23.8% (n=19). The mean age of participants was 40.7 ± 13.4 years (median 40.0 years), with the majority falling in the 31-50-year age group. The mean body mass index (BMI) was 24.37 ± 4.77 kg/m² (median 23.80 kg/m²), with 37.5% of patients classified as overweight or obese and 52.5% having normal weight.

The distribution of ASA physical status classification showed that 67.5% (n=54) were ASA Grade I, 27.5% (n=22) were ASA Grade II, and 5.0% (n=4) were ASA Grade III. Despite observed demographic variations between the two groups, there were no statistically significant differences in baseline characteristics, including age (40.8 ± 14.7 vs. 40.6 ± 12.1 years, p=0.566), BMI (24.7 ± 4.6 vs. 24.1 ± 4.9 kg/m², p=0.476), gender distribution (p=0.60), and ASA grade (p=0.88), confirming successful randomization (Table 1).

**Table 1:** Comparison of the baseline data between the two groups (n=80). ASA = American Society of Anaesthesiologists.

Characteristic	Body-First (n=40)	Conventional (n=40)	p-value
Age (years), mean ± SD	40.8 ± 14.7	40.6 ± 12.1	0.566
Age 18–30 years, n (%)	14 (35.0)	11 (27.5)	
Age 31–45 years, n (%)	16 (40.0)	18 (45.0)	
Age 46–60 years, n (%)	7 (17.5)	9 (22.5)	
Age >60 years, n (%)	3 (7.5)	2 (5.0)	
Male sex, n (%)	11 (27.5)	8 (20.0)	0.600
Female sex, n (%)	29 (72.5)	32 (80.0)	
BMI (kg/m²), mean ± SD	24.7 ± 4.6	24.1 ± 4.9	0.476
Underweight, n (%)	3 (7.5)	5 (12.5)	
Normal BMI, n (%)	21 (52.5)	21 (52.5)	
Overweight, n (%)	11 (27.5)	10 (25.0)	
Obese Class I, n (%)	3 (7.5)	2 (5.0)	
Obese Class II, n (%)	2 (5.0)	2 (5.0)	
ASA I, n (%)	28 (70.0)	26 (65.0)	0.880
ASA II, n (%)	10 (25.0)	12 (30.0)	
ASA III, n (%)	2 (5.0)	2 (5.0)	

Pre-operative clinical and ultrasonographic parameters

Pre-operative clinical history revealed that 61.3% (n=49) of patients had a history of acute cholecystitis, with similar proportions in both groups (62.5% in body-first vs. 60.0% in conventional, p=0.81). A history of chronic cholecystitis was present in 10.0% of patients in both groups (p=1.00). Previous abdominal surgery was documented in 38.7% (n=31) of patients, with no significant difference between groups (37.5% vs. 40.0%, p=0.821). Ultrasonography parameters demonstrated that 80.0% (n=64) of patients had normal gallbladder wall thickness (<3 mm), while 13.8% (n=11) had wall thickening (≥3 mm). Contracted gallbladder was observed in 11.3% (n=9) of cases, with a trend towards higher incidence in the body-first group (17.5% vs. 5.0%, p=0.079). Multiple stones were present in 61.3% (n=49) of patients, and stone impaction at the neck or cystic duct was noted in 8.8% (n=7). Common bile duct (CBD) diameter was normal in 97.5% (n=78) of patients, with only one patient showing dilation. None of the ultrasonographic parameters showed statistically significant differences between the two groups.

Biochemical Parameters

Pre-operative biochemical evaluation showed that 91.3% (n=73) of patients had normal white blood cell (WBC) counts (<11.0 × 10³/µL), with 8.8% (n=7) showing elevated counts. C-reactive protein (CRP) levels were normal (<6.0 mg/L) in 60.0% (n=48) of patients, with 40.0% (n=32) showing elevation. Liver function tests, including serum glutamic-oxaloacetic transaminase (SGOT), serum glutamic-pyruvic transaminase (SGPT), alkaline phosphatase (ALP), total bilirubin, and bilirubin fractions, remained within normal limits in the majority of patients. Normal ALP levels (<128 U/L) were observed in 76.3% (n=61) of patients, while 23.8% (n=19) had elevated values. No statistically significant differences were observed between the body-first and conventional approach groups for any of the biochemical parameters evaluated.

Primary outcomes

Achievement of Critical View of Safety

The primary outcome of this study was the rate of CVS achievement, assessed using standardized doublet photography and blinded evaluation. Among patients in the body-first approach group, 92.5% (37/40) had satisfactory CVS, compared with 75.0% (30/40) in the conventional approach group. Unsatisfactory outcomes were noted in 7.5% (3/40) and 25.0% (10/40) of patients, respectively. This inter-group difference was statistically significant (χ²=4.50, p=0.034).

Duration of Surgery

The mean operative time for all procedures was 80.5 ± 32.98 minutes (median 67.0 minutes), with the body-first group averaging 76.3 ± 27.6 minutes and the conventional group 84.8 ± 37.4 minutes. There was no statistically significant difference in operative duration between the two groups (U=682.5, Z=−0.775, p=0.438) (Table 2).

**Table 2:** Pre- and post-operative outcomes (n=80) U = Mann‑Whitney U test; Z = standardized test statistic from Mann‑Whitney U. p‑value < 0.05 indicates statistical significance. IQR = interquartile range.

Outcome	Body-First (n=40), Median (IQR)	Conventional (n=40), Median (IQR)	P (Mann–Whitney U)	Effect Size (r or Z)	Cohen’s d
Duration of surgery (min)	67.5 (83.3-59)	68.5 (101-50)	0.438	r = 0.12	d=0.086
Pre-operative CRP (mg/L)	5.71 (6.94-5.11)	5.6 (6.38-5.18)	0.949	r = 0.01	d=0.2389
Post-operative CRP (mg/L)	14.9 (28.2-7.73)	15.4 (36.5-7.45)	0.648	r = 0.05	d=0.2775

Secondary outcomes

Operative Difficulty and Conversion to Open Surgery

Operative difficulty was assessed using the Modified Nassar grading system. In the body-first group, Grade I difficulty was observed in 40.0% (n=16), Grade II in 25.0% (n=10), Grade III in 22.5% (n=9), Grade IV in 12.5% (n=5), and Grade V in 0% (n=0). In the conventional group, Grade I was observed in 35.0% (n=14), Grade II in 27.5% (n=11), Grade III in 15.0% (n=6), Grade IV in 22.5% (n=9), and Grade V in 0% (n=0). Overall, 37.5% (n=30) of cases were Grade I, 26.3% (n=21) Grade II, 18.8% (n=15) Grade III, 17.5% (n=14) Grade IV, and no patients were classified as Grade V (0%, n=0).

Univariate analysis demonstrated a significant association between operative difficulty, assessed using the Modified Nassar grading system, and achievement of the Critical View of Safety (CVS) (Fisher's exact test, p<0.001). The rate of satisfactory CVS achievement progressively declined with increasing operative difficulty, from 96.7% in Grade I and 100% in Grade II to 86.7% in Grade III and 28.6% in Grade IV, indicating that greater operative difficulty was strongly associated with failure to achieve satisfactory CVS. Conversion to open surgery occurred in 3.8% (3/80) of all patients, with 2.5% (1/40) in the body-first group and 5.0% (2/40) in the conventional group. This difference was not statistically significant (p=0.556). The converted patient in the body-first group had a frozen Calot's triangle and underwent a fenestrating subtotal cholecystectomy as a bailout procedure. No subtotal cholecystectomy was performed in the conventional group.

Intraoperative Complications

Intraoperative complications were minimal across both groups. Minor bleeding occurred in 20.0% (n=8) of patients in the body-first group and in 17.5% (n=7) in the conventional group. No major complications such as bile duct injury, bowel injury, or significant hemorrhage requiring blood transfusion were observed in either group. The difference in complication rates between groups was not statistically significant (p >0.05) (Table 3).

**Table 3:** Categorical post-operative outcomes (n=80) χ² = Pearson Chi-Square; OR=odds ratio; CI=confidence interval; p‑value < 0.05 indicates statistical significance.

Outcomes	Body-First (n=40), n (%)	Conventional (n=40), n (%)	p-value (test)	Effect size (OR,95% CI)
CVS achieved	37 (92.5%)	30 (75.0%)	0.066 (Fisher's exact)	4.11 (1.04-16.3)
GI function recovery <24 hrs.	27 (67.5%)	16 (40.0%)	0.014 (χ²)	3.12 (1.25-7.75)
Post-operative pain	15 (37.5%)	31 (77.5%)	<0.001 (χ²)	5.74 (2.15-15.3)
Intra-operative complications	8 (20.0%)	7 (17.5%)	0.775 (χ²)	0.848 (0.275-2.61)
Post-operative complications	2 (5.0%)	3 (7.5%)	0.644 (χ²)	0.649 (0.10-4.11)

Postoperative Pain

Postoperative pain assessment using a visual analog scale (VAS) demonstrated significantly lower pain scores in the body-first approach group. This difference was statistically significant (χ²=13.06, p=0.001). Median VAS score was 0 (IQR 0-3) in the body-first group compared with 3.5 (IQR 1-5) in the conventional group (Mann-Whitney U = 457, p<0.001). Mean pain scores were 1.50±2.31 and 3.15±2.33, respectively. The observed difference demonstrated a moderate-to-large effect size (rank biserial correlation=0.43; Cohen’s d=0.71) (Table 3).

Gastrointestinal Function Recovery

Recovery of gastrointestinal function, measured by the passage of first flatus, was categorized as within 24 hours versus beyond 24 hours. In the body-first group, 67.5% (n=27) of patients recovered gastrointestinal function within 24 hours compared to 40.0% (n=16) in the conventional group. This difference was statistically significant (OR: 3.12, 95% CI: 1.25-7.75, p=0.014) (Table 3).

Postoperative C-Reactive Protein Levels

Postoperative CRP levels measured 24 hours after surgery showed no significant difference between the two groups. In the body-first group, 72.5% (n=29) had normal CRP levels (<6.0 mg/L) and 27.5% (n=11) had elevated levels. In the conventional group, 80.0% (n=32) had normal levels and 20.0% (n=8) had elevated levels (p=0.648) (Table 2).

Postoperative Nausea and Vomiting

Postoperative nausea and vomiting were less common in the body-first group, with 87.5% (n=35) reporting no symptoms compared to 80.0% (n=32) in the conventional group. However, this difference did not reach statistical significance (p>0.05).

Health-Related Quality of Life

Health-related quality-of-life assessment using the RAND-36 demonstrated superior outcomes with the body-first approach, particularly in the Physical Functioning domain, which remained significantly higher at discharge (p<0.001; Mann-Whitney U=431.5), one month (p<0.001; Mann-Whitney U=402.5), and three months (p<0.001; Mann-Whitney U=258.5). Energy/Fatigue scores also favored the body-first group at discharge (p=0.029; Mann-Whitney U=585.0), one month (p=0.014; Mann-Whitney U=515.0), and three months (p=0.004; Mann-Whitney U=475.0). While Role-Physical, Role-Emotional, and Emotional Well-being remained comparable between groups, later improvements favoring the body-first approach were observed in Social Functioning (three months: p=0.034; Mann-Whitney U=558.0), Pain (one month: p=0.023, Mann-Whitney U=540.0; three months: p=0.003, Mann-Whitney U=455.0), and General Health (three months: p=0.049; Mann-Whitney U=590.5).

Multivariable Analysis

Before multivariable analysis, multicollinearity was assessed using variance inflation factors (VIF) and tolerance statistics. All predictors demonstrated acceptable VIF and tolerance values, indicating no evidence of significant multicollinearity. Given the limited number of unsatisfactory CVS events, the multivariable model was restricted to two prespecified clinically relevant predictors: surgical technique and operative difficulty (Modified Nassar grade) to minimize overfitting and maintain an adequate events-per-variable ratio. Multivariable logistic regression identified both surgical technique and operative difficulty as independent predictors of satisfactory Critical View of Safety (CVS) achievement. Compared with the body-first approach, the conventional technique was associated with significantly lower odds of achieving satisfactory CVS (adjusted OR 0.10, 95% CI 0.02-0.66; p = 0.017). Increasing operative difficulty, assessed using the Modified Nassar grade, was independently associated with reduced odds of satisfactory CVS achievement (adjusted OR 0.11 per one-grade increase, 95% CI 0.03-0.45; p = 0.002). The model demonstrated good fit, with a deviance of 37.6, an Akaike Information Criterion (AIC) of 43.6, and a McFadden pseudo-R² of 0.470.

Multiple linear regression was performed to identify independent predictors of operative duration. The overall model demonstrated a moderate fit (R = 0.532, R² = 0.283, adjusted R² = 0.234), explaining 28.3% of the variability in operative duration. After adjustment for age, body mass index, surgical technique, operative difficulty, and previous acute cholecystitis, increasing operative difficulty, assessed using the Modified Nassar grade, was the only independent predictor of longer operative duration (β = 13.32 minutes per one-grade increase, SE = 3.12; 95% CI 7.11-19.53; p < 0.001). Age (β = 0.18 minutes/year, SE = 0.26; p = 0.505), BMI (β = 0.97 minutes/kg/m², SE = 0.70; p = 0.172), surgical technique (β = 6.54 minutes for the conventional versus body-first approach, SE = 6.48; p = 0.316), and previous acute cholecystitis (β = 3.25 minutes, SE = 6.74; p = 0.631) were not independently associated with operative duration) (Table 4).

**Table 4:** Multivariable regression analysis for duration of surgery (n=80) OR = Odds ratio; CI = Confidence interval; β = Regression coefficient; SE = Standard errors; BMI = Body mass index.

Predictor	Duration of Surgery (Linear Regression) β (SE)	p-value
Age	0.177 (0.264)	0.505
BMI	0.970 (0.703)	0.172
Conventional vs Body-first approach	6.544 (6.480)	0.316
Operative difficulty (Modified Nassar grade)	13.319 (3.117)	<0.001
Acute Cholecystitis (Yes vs No)	3.251 (6.740)	0.631

Sensitivity Analysis

Sensitivity analyses were performed to assess the robustness of the primary outcome. In the intention-to-treat analysis, satisfactory CVS achievement remained higher in the body-first group than in the conventional group (92.5% vs. 75.0%; OR 4.11, 95% CI 1.04-16.29). Fisher's exact test was used because one expected cell count was less than five, yielding a two-sided p-value of 0.066. Although this did not meet the conventional threshold for statistical significance, the direction and magnitude of the treatment effect remained consistent, supporting the robustness of the observed association. Per-protocol analysis, excluding converted cases, showed a similar finding, with satisfactory CVS achieved in 94.9% of patients in the body-first group compared with 78.9% in the conventional group (p=0.047). Furthermore, multivariable logistic regression confirmed surgical technique as an independent predictor of CVS achievement, with the conventional approach associated with significantly lower odds of achieving satisfactory CVS compared with the body-first approach (adjusted OR 0.10, 95% CI 0.02-0.66; p=0.017). Collectively, these analyses support the robustness and consistency of the primary findings.

Subgroup Analysis

**Figure 5:**
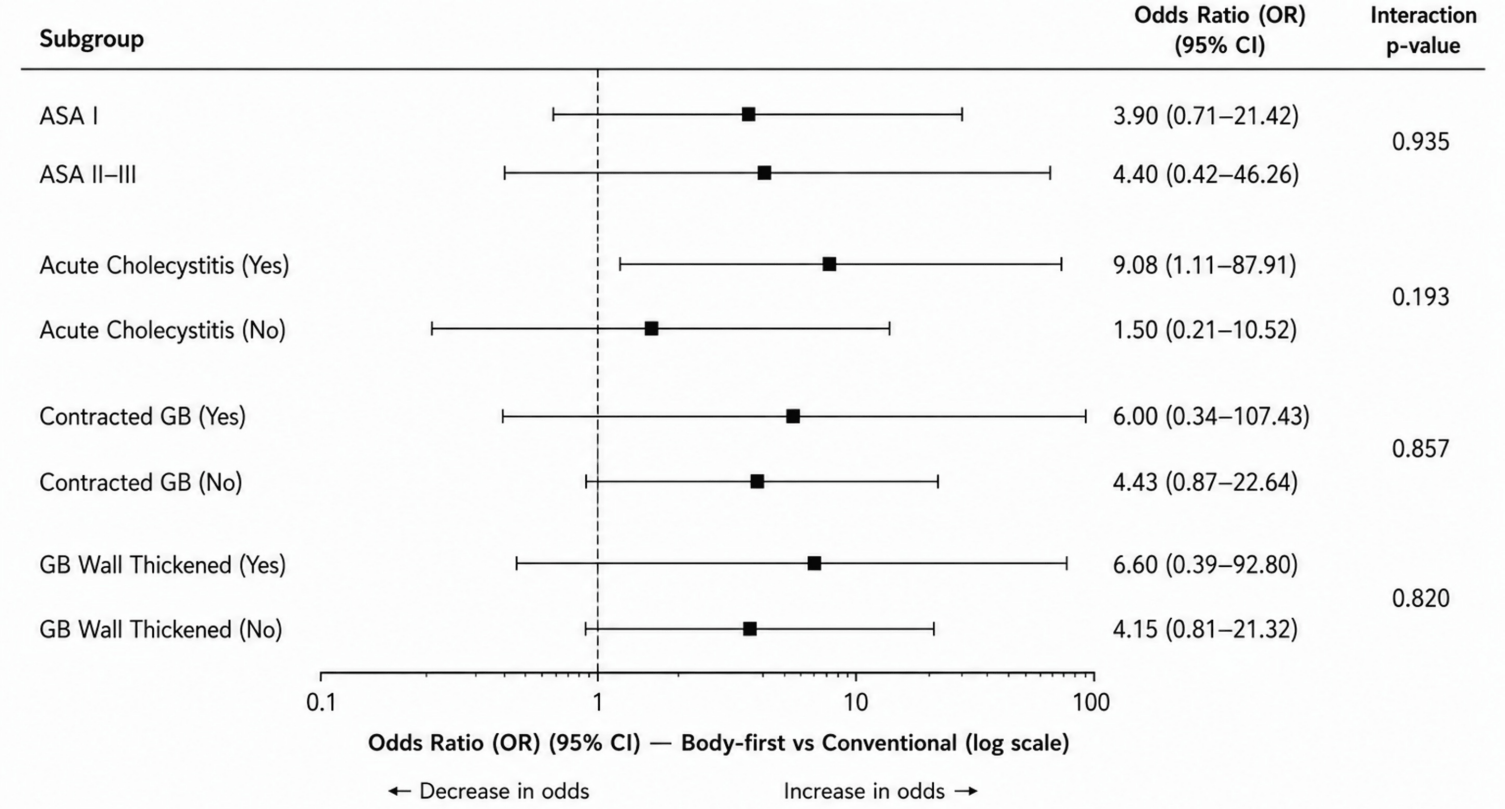
Forest plot for subgroup analysis of odds ratios Forest plot showing subgroup analysis of odds ratios (OR) for successful achievement of Critical View of Safety (CVS) comparing body-first versus conventional laparoscopic cholecystectomy. Horizontal bars represent 95% confidence intervals. The vertical reference line at OR = 1 indicates no difference between groups. ASA = American Society of Anaesthesiologists, GB = Gall bladder.

The body-first approach showed higher CVS achievement across most subgroups; however, differences were not statistically significant, and interaction tests were non-significant. Outcomes were comparable in ASA II-III patients (Figure 5).

Overall, the benefit of the body-first technique was consistent across clinical subgroups, with no significant heterogeneity (Table 5).

**Table 5:** Subgroup analysis of CVS achievement (n=80) OR = odds ratio; CI = confidence interval; p‑value < 0.05 indicates statistical significance, ASA = American Society of Anaesthesiologists.

Variable	Category	Body-First CVS n (%)	Conventional CVS n (%)	OR (95% CI)	Interaction p
ASA Grade	ASA I	26/28 (92.9%)	20/26 (76.9%)	3.90 (0.71–21.42)	0.935
	ASA II–III	11/12 (91.7%)	10/14 (71.4%)	4.40 (0.42–46.26)	
Acute Cholecystitis	Yes	24/25 (96.0%)	17/24 (70.8%)	9.88 (1.11–87.91)	0.193
	No	13/15 (86.7%)	13/16 (81.3%)	1.50 (0.21–10.52)	
Contracted Gallbladder	Yes	6/7 (85.7%)	2/4 (50.0%)	6.00 (0.34–107.43)	0.857
	No	31/33 (93.9%)	28/36 (77.8%)	4.43 (0.87–22.64)	
Gallbladder Wall Thickening	Yes	9/10 (90.0%)	3/5 (60.0%)	6.00 (0.39–92.28)	0.820
	No	28/30 (93.3%)	27/35 (77.1%)	4.15 (0.81–21.32)	

## Discussion

The prevention of bile duct injury remains a primary objective in LAP-C. Although its incidence is relatively low, the clinical and economic burden of BDI is substantial [4,5]. Evidence consistently indicates that most injuries arise from visual misperception and anatomical misidentification rather than from technical error alone [4]. Consequently, the routine achievement and documentation of the CVS have become central to safe cholecystectomy practice [6-8].

More recently, Nassar et al. emphasized predictors of failure to achieve CVS and highlighted the importance of alternative dissection strategies in complex cases [5]. International consensus guidelines published within the last five years further reinforce the need for structured strategies, including bailout techniques and fundus-first dissection when standard exposure is unsafe [7,8]. Matsumura et al. reported improved visualization and favorable safety outcomes using a body-first strategy in difficult gallbladders [11]. Tuveri and Tuveri described the dome-down approach as advantageous in fibrotic or contracted gallbladders [12]. Similarly, Ohtani et al. retrospectively analyzed the usefulness of the body-first approach in difficult laparoscopic cholecystectomy [13].

The present RCT is, to our knowledge, one of the first prospective randomized trials to compare body-first and conventional LAP-C techniques with CVS achievement as the primary endpoint, objectively verified by blinded doublet photography. Our central finding that the body-first technique achieved satisfactory CVS in 92.5% of patients, compared with 75.0% with the conventional approach (p=0.066), provides evidence of a clinically meaningful safety advantage of the retrograde dissection strategy. The adjusted odds ratio of 0.10 for the conventional technique demonstrates that the body-first approach remained an independent predictor of satisfactory CVS achievement after adjustment for operative difficulty. This finding suggests that the improved CVS achievement associated with the body-first technique extends beyond differences in operative complexity and reflects a true technique-related advantage.

By commencing dissection at the gallbladder body and advancing retrogradely, the surgeon establishes clear anatomical landmarks in a zone inherently less affected by pericholecystic inflammation before approaching the hepatocystic triangle. This stands in contrast to the conventional antegrade technique, which immediately enters the most inflamed and anatomically ambiguous region. As articulated by Pucher et al., visual-perceptual errors are a major contributor to vasculobiliary injury during laparoscopic cholecystectomy, especially when inflammation or distorted anatomy compromises the accurate identification of structures within the hepatocystic triangle. This provides a rationale for alternative dissection strategies that avoid early dissection in a hostile Calot's triangle [18]. The body-first technique essentially builds in a margin of anatomical orientation before the critical structures are engaged.

Our findings align with and extend observations from several prior reports. Matsumura et al. demonstrated improved anatomical exposure using subserosal retrograde dissection [11]. Tuveri and Tuveri reported that the dome-down approach was particularly advantageous in fibrotic or contracted gallbladders, a subgroup well-represented in our cohort [12]. Most compellingly, Zhang et al. found that Laennec's membrane-guided body-first LAP-C for acute cholecystitis was associated with lower blood loss, faster GI recovery, reduced CRP elevation, shorter hospital stays, and zero BDI, a profile that closely mirrors our own findings despite the difference in patient acuity [14]. A concern frequently raised about alternative dissection techniques is the prospect of prolonged operative time, which carries its own risks, such as prolonged anesthesia, increased thermal injury, and surgeon fatigue.

Despite concerns that the body-first approach may prolong surgery, operative duration was comparable between the two groups (76.3 vs. 84.8 minutes; p = 0.438). Moreover, multivariable analysis demonstrated that surgical technique was not an independent predictor of operative duration (β = 6.54 minutes, p = 0.316). Instead, increasing operative difficulty emerged as the only independent determinant, with each one-grade increase in Modified Nassar grade associated with an additional 13.32 minutes of operative time (p < 0.001). These findings suggest that operative complexity, rather than the choice of dissection technique, primarily influences surgical duration.

Operative difficulty exerted a substantial influence on CVS achievement. The proportion of satisfactory CVS progressively declined with increasing Modified Nassar grade, decreasing from 96.7% in Grade I and 100% in Grade II to 86.7% in Grade III and only 28.6% in Grade IV. This finding is consistent with the established understanding that severe inflammation, dense adhesions, fibrosis, and distortion of Calot’s anatomy in higher Nassar grades make safe dissection increasingly challenging [5]. Notably, operative difficulty remained an independent predictor of CVS achievement on multivariable analysis, highlighting the importance of preoperative risk stratification and adoption of alternative dissection strategies in technically demanding cases. The postoperative benefits observed in the body-first group merit attention. Significantly lower visual analog scale (VAS) pain scores (p<0.001) and faster gastrointestinal recovery (p=0.014). Although postoperative CRP levels were lower in the body-first group, the difference was not statistically significant, possibly reflecting reduced visceral manipulation.

The quality-of-life findings complemented the clinical outcome data, with the body-first group demonstrating significantly higher RAND-36 Physical Functioning scores at discharge (p<0.001; Mann-Whitney U=431.5), one month (p<0.001; Mann-Whitney U=402.5), and three months (p<0.001; Mann-Whitney U=258.5), along with superior Energy/Fatigue scores at discharge (p=0.029; Mann-Whitney U=585.0), one month (p=0.014; Mann-Whitney U=515.0), and three months (p=0.004; Mann-Whitney U=475.0). Additional improvements favoring the body-first approach were observed in Pain at one and three months, and in Social Functioning and General Health at three months. These findings suggest that the benefits of body-first dissection extend beyond operative outcomes to improved patient-centered recovery. From the patient’s perspective, earlier restoration of physical function and vitality, and a return to normal daily activities, are among the most meaningful indicators of postoperative recovery [17].

The multivariable analyses demonstrated that increasing operative difficulty, assessed using the Modified Nassar grade, was the principal independent determinant of both CVS achievement and operative duration. Each one-grade increase in operative difficulty was associated with an 89% reduction in the odds of achieving satisfactory CVS (adjusted OR 0.11, 95% CI 0.03-0.45; p=0.002) and an increase of approximately 13.3 minutes in operative duration (β=13.32 minutes per grade increase, 95% CI 7.11-19.53; p<0.001). Despite adjustment for operative difficulty, the conventional technique remained independently associated with significantly lower odds of achieving satisfactory CVS than the body-first approach (adjusted OR 0.10, 95% CI 0.02-0.66; p=0.017), indicating that the advantage of the body-first technique extends beyond differences in operative complexity. These findings are consistent with previous reports demonstrating that increasing operative difficulty, characterized by inflammation, fibrosis, and distorted biliary anatomy, is a major determinant of successful CVS achievement and operative complexity [8,9].

Limitations

A few limitations deserve acknowledgment. First, blinding of the operating surgeon was not possible for an inherent reason: the intervention is defined by the surgical technique itself. However, the critical outcome, CVS quality, was assessed by blinded evaluators using standardized photographic documentation, substantially mitigating this limitation. Second, the study was conducted at a single high-volume tertiary center with experienced surgeons trained in both techniques; generalisability to lower-volume or community settings where body-first experience may be limited requires caution. Third, longer-term follow-up beyond three months is warranted to assess any divergence in quality-of-life trajectories and late complications. Fourth, while the sample size was adequate for the primary endpoint, the study may have been underpowered to detect small differences in rare outcomes such as BDI, which did not occur in either group. Finally, the body-first approach requires a defined learning curve; surgeons introducing this technique de novo may not immediately replicate these outcomes.

As with all operative studies, patient-specific anatomical and inflammatory variations may influence the precise conduct of dissection. Accordingly, reproducibility should be viewed as the consistent application of a standardized surgical strategy rather than replication of identical intraoperative conditions. Operative difficulty was prospectively graded using the Modified Nassar classification to account for this variability.

Future multicentre randomized trials with larger cohorts and longer follow-up are required to determine whether improved CVS achievement translates into measurable reductions in bile duct injury at the population level. Integration of the body-first technique into structured laparoscopic training curricula may also warrant evaluation. In summary, the body-first approach improves the rate of CVS achievement without increasing operative time and is associated with reduced postoperative pain and faster recovery. These findings support its consideration as a safe and effective alternative to conventional Calot’s triangle-first dissection, particularly in anatomically challenging cases.

## Conclusions

This study demonstrates that the body-first dissection technique achieves a significantly higher rate of satisfactory CVS compared to the conventional approach during elective LAP-C. Beyond the primary safety endpoint, patients in the body-first group experienced less postoperative pain and a faster return of gastrointestinal function. These clear advantages make body-first dissection a practical, safer option for surgeons to achieve CVS while performing LAP-C. Routine training in this method could meaningfully improve patient outcomes in gallbladder surgery.
